# Chemsex and rising substance use linked to sexually transmitted infections among men who have sex with men living with HIV in Bangkok, Thailand

**DOI:** 10.1016/j.ijregi.2024.100465

**Published:** 2024-09-28

**Authors:** Camilla Muccini, Suteeraporn Pinyakorn, Christy Kolsteeg, Eugène Kroon, Carlo Sacdalan, Trevor A. Crowell, Phillip Chan, Robert Paul, Denise Hsu, Nittaya Phanuphak, Donn J. Colby, RV254/SEARCH010 Study Group

**Affiliations:** 1 Department of Infectious Diseases, IRCCS San Raffaele Scientific Institute, Milan, Italy; 2 SEARCH Research Foundation, Bangkok, Thailand; 3 United States Military HIV Research Program, Center for Infectious Disease Research, Walter Reed Army Institute of Research, Silver Spring, MD, USA; 4 Henry M. Jackson Foundation for the Advancement of Military Medicine, Bethesda, MD, USA; 5 University Medical Center Utrecht, Utrecht, The Netherlands; 6 Research Affairs, Faculty of Medicine, Chulalongkorn University, Bangkok, Thailand; 7 Department of Neurology, Yale School of Medicine, New Haven, CT, USA; 8 Yale Center for Brain & Mind Health, Yale University, New Haven, CT, USA; 9 Missouri Institute of Mental health, University of Missouri–St. Louis, St. Louis, MO, USA; 10 Institute of HIV Research and Innovation, Bangkok, Thailand

**Keywords:** HIV, Antiretroviral therapy, Substance use, Sexually transmitted diseases, Southeastern Asia

## Abstract

**Objectives:**

We report longitudinal trends in alcohol and recreational drug use, and their associations with sexual behaviors and clinical outcomes in a Thai cohort of predominantly men who have sex with men (MSM) living with HIV.

**Methods:**

From 2017 to 2019, participants in the RV254/SEARCH010 acute HIV cohort answered questions every 24 weeks about drug use and sexual behaviors. Longitudinal trends were assessed using the χ2 test for trend. Logistic regression was used to estimate odds ratios (ORs) and 95% confidence intervals (CIs) for factors potentially associated with recreational drug and alcohol use.

**Results:**

Among 604 participants, the median age was 26 (interquartile range 23-31) years, and 93.5% were MSM. Alcohol consumption was reported in 83.3% and recreational drug use in 46.9% during the study period, with rising trends in both over the years. Participants who reported recreational drug use were more likely to have hepatitis C (OR 3.42, 95% CI 1.88-6.21), syphilis (OR 2.69, 1.75-4.13), gonorrhea (OR 7.74, 5.04-11.89), and chlamydia (OR 1.61, 1.12-2.31), and to engage in group sex (OR 7.74, 5.04-11.89). Participants who used any recreational drugs had more frequent viral blips (23.1% vs 14.2%, *P* = 0.007) and reported missed doses of antiretroviral drugs more often (52.4% vs 36.9%, *P* <0.001), but had no significant difference in viral suppression (94.1% vs 97.4%, *P* = 0.06).

**Conclusions:**

Among MSM living with HIV in Bangkok, recreational drug use has increased in recent years and was associated with hepatitis C and sexually transmitted infections, especially among men who participate in group sex. Prevention strategies and other interventions may improve treatment adherence and other HIV outcomes.

## Highlights

• Recreational drug use increased among men who have sex with men with HIV in Bangkok.
• Higher rates of hepatitis C virus and sexually transmitted infections were linked to group sex.
• Substance use affects adherence to treatment and the occurrence of viral blips.
• These data provide insights for targeted public health interventions in HIV.

## Introduction

Thailand is one of the countries of the “Golden Triangle,” where the production of synthetic drugs has dramatically increased and drug trafficking has expanded over the past few years [1]. Starting from the early 2000s, methamphetamine has become the most common illicit drug consumed among Thai people, followed by heroin and ketamine. The number of crystal methamphetamine users receiving substance use treatment increased from 8398 in 2016 to 16,381 in 2019 [2,3]. A similar trend was observed in other Southeast Asian countries, including Vietnam and Laos [4,5]. Among young Thai men who have sex with men (MSM) in Bangkok, the observed surge of methamphetamine consumption has been associated with a greater incidence of HIV, commercial sex work, group sex, and use of dating applications [6].

Worldwide, drug use among MSM is typically higher than in the general population [7]. The intentional use of recreational drugs before or during encounters to enhance or prolong sexual experiences, termed chemsex in Western countries or hi-fun in Southeast Asia, is also increasing among MSM [8]. In addition, compared with MSM without HIV, MSM with HIV have a greater prevalence of recreational drug use, chemsex, polysubstance use [9], and mental health challenges such as depression and anxiety [8]. Substance use can trigger sexual behaviors that increase the risk of contracting sexually transmitted infections (STIs), including condomless intercourse and multiple sexual partners [10]. As a result, HIV and other STIs have been rising among MSM reporting substance and alcohol use [11].

We have previously reported a surge in hepatitis C virus (HCV) incidence among MSM living with HIV in Bangkok that was associated with methamphetamine use [12] and a history of syphilis [13]. HIV, STIs, viral hepatitis, and substance use are often considered elements of a syndemic, defined as a combination of inter-related and mutually reinforcing health issues that together create an excess burden of disease in a population [14]. A better understanding of this syndemic is needed in low- and middle-income countries. It is also crucial to evaluate the impact of substance use on adherence to antiretroviral therapy (ART) and viral suppression in people treated with an effective regimen. The aim of these analyses was to assess longitudinal trends in alcohol and recreational drug use and to determine sexual behaviors, ART adherence, and virologic outcomes associated with substance use in a Thai cohort of MSM living with HIV.

## Methods

The RV254/SEARCH010 study (NCT00796146) is a prospective cohort that has been enrolling adults with acute HIV in Bangkok, Thailand since 2009 [15]. Participants are offered ART initiation under a separate protocol (NCT00796263) and are followed every 12-24 weeks thereafter.

Substance use over the previous 4 months was recorded at the enrollment visit. Starting from 2017, a computer-based questionnaire about drug, alcohol use, and sexual behavior was completed every 24 weeks after enrollment. Substance use data included alcohol, inhaled alkyl nitrites (poppers), amphetamine, methamphetamine, opium, heroine, ecstasy, ketamine, gamma-hydroxybutyrate, marijuana, cocaine, and non-prescribed use of erectile dysfunction medications. Substance use was defined as self-reported use of one or more substances during a calendar year; the highest frequency recorded was considered for each participant. Risky alcohol use was defined by an AUDIT-C (Alcohol Use Disorders Identification Test) score ≥4 [16]. Recreational drug use was defined as the use of any substance other than alcohol or erectile dysfunction drugs.

Screening for HCV antibodies and for *Neisseria gonorrhoeae* and *Chlamydia trachomatis* using nucleic acid amplification testing was performed at enrollment and then annually. Screening for syphilis using *Treponema pallidum* hemagglutination assay (TPHA) with rapid plasma reagin (RPR) confirmation was conducted every 24 weeks. Syphilis incidence was defined as (1) both TPHA and RPR tests positive in a participant without a history of syphilis, (2) a four-fold increase in RPR titer, or (3) seroconversion from non-reactive to reactive RPR in a participant with a history of syphilis. Participants with RPR positive at week 0 were not counted as incidence cases. STI testing was performed more frequently on an individual basis when clinically indicated.

HIV RNA testing was performed every 12-24 weeks using the COBAS TaqMan HIV-1 Test v2.0 (Roche Molecular Systems), with the lower limit of quantification at 20 copies/ml. A viral blip was defined as any HIV RNA of 20-999 copies/ml immediately preceded and followed by HIV RNA of <20 copies/ml. Adherence to ART was assessed by participant self-report at each visit.

All participants with substance use data collected between September 5, 2017 and December 31, 2019 were included in these analyses. Results were reported as medians with interquartile range (IQR) or frequency (%). Drug use by calendar year was compared using the χ2 test for trend. Participant characteristics were compared using the Mann–Whitney U or Fisher's exact tests, as appropriate. Logistic regression was used to estimate odds ratios (ORs) and 95% confidence intervals (CIs) for factors associated with recreational drug and risky alcohol use. All tests were two-sided, with a significance level of 5%. All statistical analyses were performed using Stata 16.0 (StataCorp LLC, College Station, TX, USA).

All participants provided written informed consent before enrollment in the RV254/SEARCH010 study. The study protocol was approved by the institutional review boards of the Faculty of Medicine of Chulalongkorn University (Bangkok, Thailand), Walter Reed Army Institute of Research (Silver Spring, MD, USA), and all collaborating institutions.

## Results

Among 633 participants enrolled in the RV254/SEARCH010 study, we included 604 (95.4%) with substance use data collected from 2017 to 2019. During the study, 328 participants completed the substance use questionnaire in 2017, 548 in 2018, and 594 in 2019. The median age at HIV diagnosis was 26 (IQR 23-31) years, 97.7% were male, and 93.5% MSM. Overall, 306 (50.7%) had a bachelor's degree and 61 (10.1%) had a master's degree or higher. The median monthly income per participant was 40,000 (IQR 20,000-70,000) baht (approximately 1200 [IQR 600-2000] USD). By comparison, the National Statistical Office of Thailand reports that the percentage of people with a bachelor's degree or higher was 19.5% for the whole country and 34.9% for Bangkok, and the average monthly income per household 27,352 baht in 2021 [17].

Substance use by year of enrollment is reported in Table 1. At baseline, participants enrolled into the cohort between 2017 and 2019 (n = 137) were more likely than those enrolled between 2009 and 2016 to report both methamphetamine use (24.8% vs 17.1%, *P* = 0.044) and injection of methamphetamine (8.0% vs 2.1%, *P* = 0.001). Among participants enrolled between 2017 and 2019, at baseline, 104 (75.9%) reported alcohol consumption and 63 (46.0%) reported recreational drug use, including methamphetamine use in 34 (24.8%) participants.

**Table 1:** Baseline drug use by year of enrollment.

Week 0	Enrolled 2009-2016	Enrolled 2017-2019	*P*-value
N	433	137	
Any alcohol	-	104 (75.9)	
**Drug for erectile dysfunction**	**30 (6.9)**	**36 (26.3)**	**<0.001**
**Any recreational drug use**	**144 (33.3)**	**63 (46.0)**	**0.007**
**Poppers**	**58 (13.4)**	**49 (35.8)**	**<0.001**
Amphetamine-type stimulants	84 (19.4)	36 (26.3)	0.085
Ecstasy	6 (1.4)	3 (2.2)	0.511
Oral amphetamine	4 (0.9)	4 (2.9)	0.084
**Methamphetamine**	**74 (17.1)**	**34 (24.8)**	**0.044**
Methamphetamine smoking	-	13 (9.04)	
**Methamphetamine injection**	9 (2.1)	11 (8.0)	**0.001**
Opium smoking	-	-	
Heroine injection	0 (0)	1 (0.7)	0.075
Ketamine	1 (0.2)	1 (0.7)	0.389
**Gamma-hydroxybutyrate**	**1 (0.2)**	**3 (2.2)**	**0.017**
**Marijuana**	**4 (0.9)**	**5 (3.7)**	**0.026**
**Cocaine**	**1 (0.2)**	**5 (3.7)**	**<0.001**

**Figure 1:**
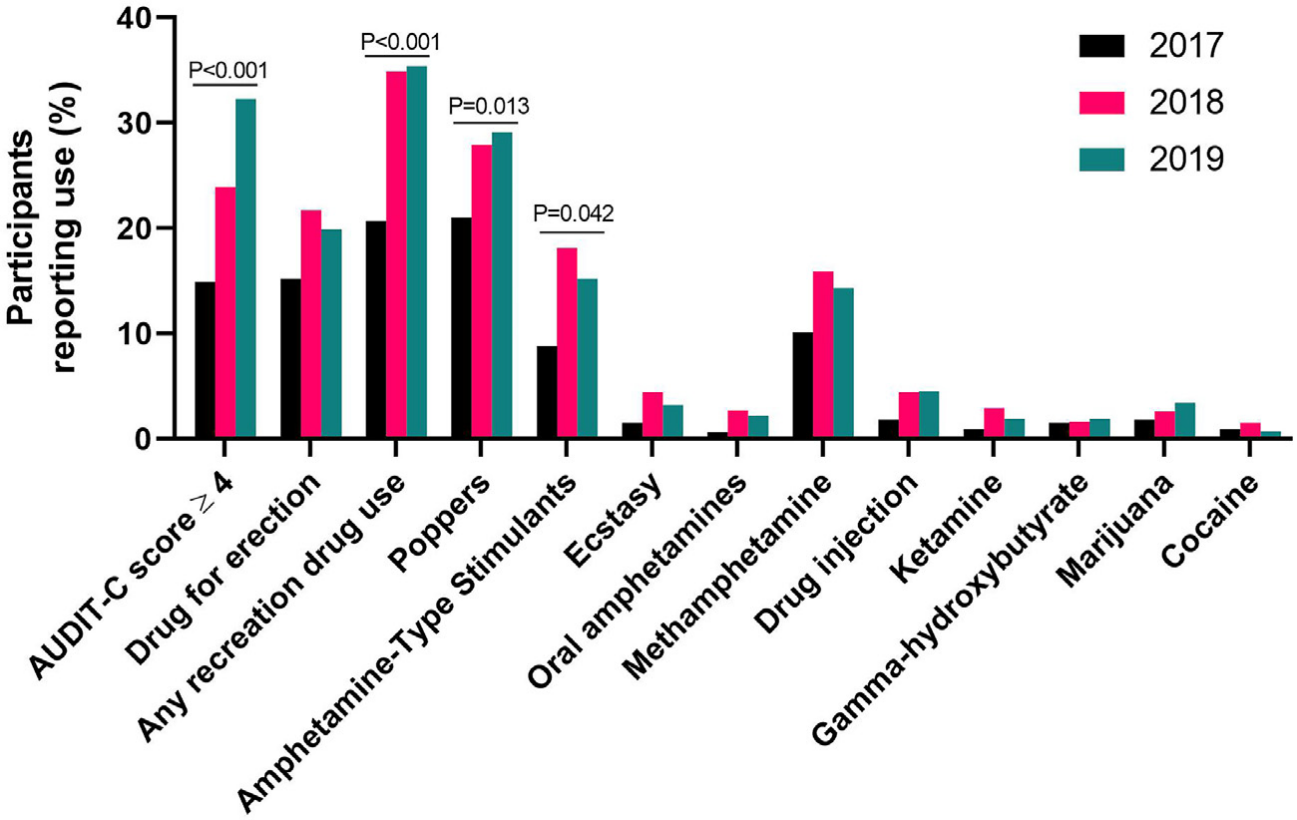
Participants reporting use of each substance by calendar year.

**Figure 2:**
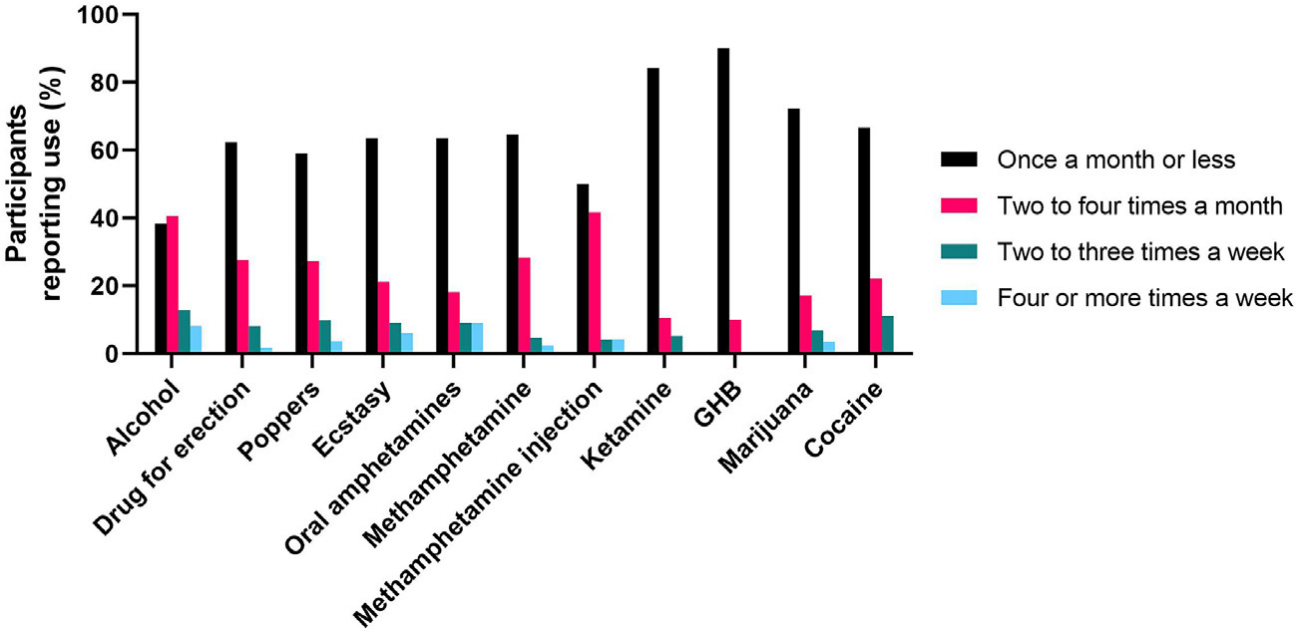
Frequency of use of each substance. GHB, gamma-hydroxybutyrate.

Figure 1 shows the proportion of cohort participants reporting use of each substance by calendar year. From 2017 to 2019, significant increases were reported in risky alcohol use, poppers, amphetamine-type stimulants, and any use of recreational drugs both at baseline and at follow-up visits. Risky alcohol use, defined as AUDIT-C score ≥4, was recorded in 230 of 604 (38.1%) participants, increasing from 49 of 328 (14.9%) in 2017 to 131 of 548 (23.9%) in 2018 and 192 (32.3%) in 2019/594 (*P* ≤0.001). Recreational drug use was reported in 283 of 604 (46.9%) participants, increasing from 68 of 328 (20.7%) in 2017 to 191 of 548 (34.9%) in 2018 and 210 of 594 (35.4%) in 2019 (*P* ≤0.001). From 2017 to 2019, the most common drugs reported were poppers (n = 242; 40.1%), erectile dysfunction drugs (n = 172; 28.5%), and methamphetamine (n = 129; 21.4%). Injection of methamphetamine and heroin was reported by 46 (7.6%) participants. Frequency of use of each substance is reported in Figure 2.

Participants who used recreational drugs at any time were more likely to have hepatitis C coinfection (OR 3.42, 95% CI 1.88-6.21, *P* ≤0.001), syphilis coinfection (OR 2.69, 95% CI 1.75-4.13, *P* ≤0.001), gonorrhea (OR 7.74, 95% CI 5.04-11.89, *P* = 0.008), chlamydia (OR 1.61, 95% CI 1.12-2.31, *P* = 0.010), and to have reported a history of group sex (OR 7.74, 95% CI 5.04-11.89, *P* ≤0.001) (Table 2, Supplemental Table 1). Methamphetamine injection was highly associated with group sex (OR 28.40, 95% CI 10.99-73.41, *P* ≤0.001), with 89% of people who inject drugs reporting use in the context of group sex events. None of the people who injected drugs reported sharing needles for injection with other people.

**Table 2:** Substance use correlation with incidence of sexually transmitted infections.

	Hepatitis C virus	Syphilis	Gonorrhea	Chlamydia	Group sex
AUDIT C ≥4	1.22 (0.71-2.10)	1.25 (0.83-1.90)	1.23 (0.83-1.83	1.34 (0.93-1.94)	**1.67 (1.16-2.40)**
Any recreational drug use	**3.42 (1.88-6.21)**	**2.69 (1.75-4.13)**	**1.70 (1.15-2.52)**	**1.61 (1.12-2.31)**	**7.74 (5.04-11.89)**
Methamphetamine use	**3.66 (2.10-6.39)**	**2.61 (1.67-4.08)**	**2.23 (1.45-3.45)**	1.52 (1.00-2.32)	**5.60 (3.69-8.50)**
Methamphetamine injection	**4.38 (2.16-8.91)**	**2.03 (1.04-3.94)**	**2.19 (1.14-4.18)**	1.78 (0.94-3.36)	**28.40 (10.99-73.41)**

Unadjusted odds ratios (95% confidence intervals) are reported. Significant associations (*P* <0.05) are bolded.

Among participants enrolled between 2017 and 2019, substance and alcohol use was assessed for associations with immunologic indicators (i.e., CD [cluster of differentiation] 4 T-cells, CD8 T-cells, CD4/CD8 T-cell ratio) at baseline and after 48 weeks of ART. The only statistically significant differences observed between those who did and did not report the use of recreational drugs or alcohol were that participants with baseline AUDIT-C score ≥4 had higher median (IQR) CD4 T-cell count at baseline (435 [327-690] vs 337 [229-456] cells/mm^3^, *P* ≤0.001) and higher median CD4/CD8 T-cell ratio at both baseline (0.8 [0.4-1.3] vs 0.6 [0.4-0.9], *P* = 0.040) and at week 48 (1.2 [0.9-1.5] vs 0.9 [0.7-1.2], *P* = 0.020).

**Figure 3:**
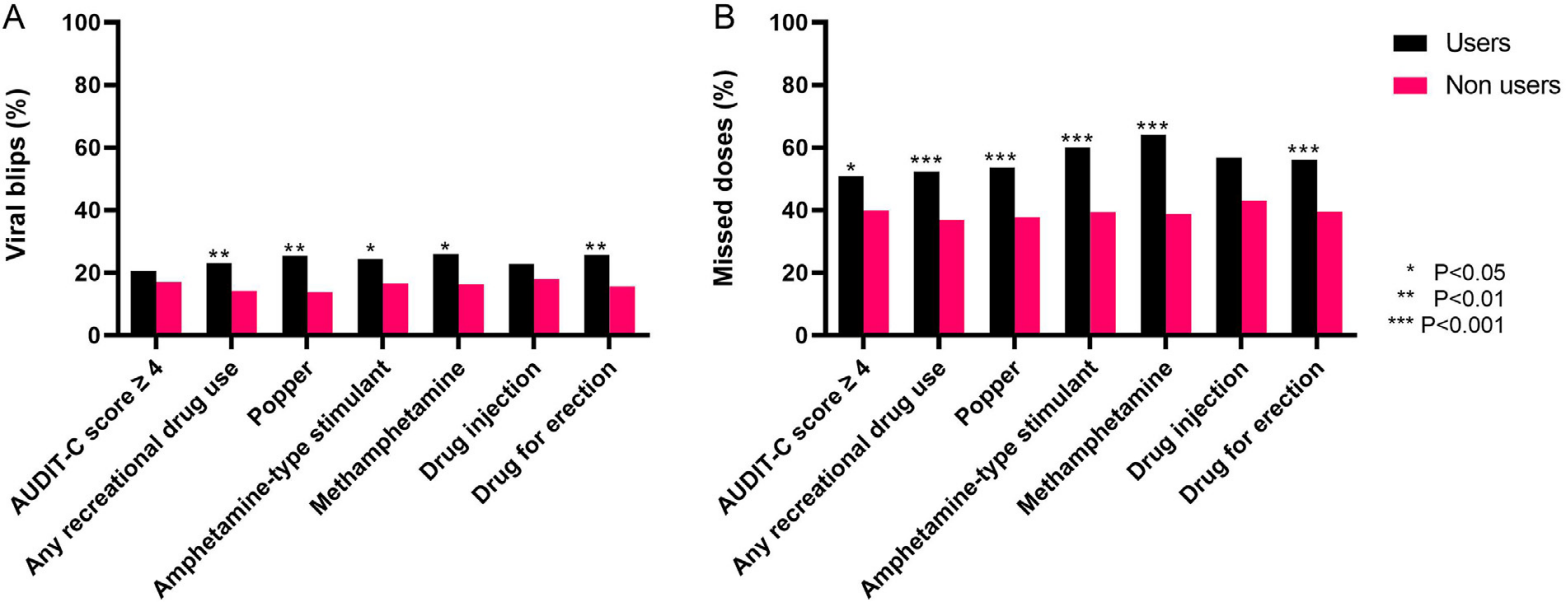
Associations between viral blips, missed doses, and the use of each substance.

Virologic outcomes were assessed among 582 participants who had completed at least 24 weeks of follow-up on ART. Participants who reported the use of poppers, methamphetamine, erectile dysfunction drugs, or “any recreational drug use” were more likely to miss doses of ART and to have viral blips (Figure 3). No significant differences in measures of ART adherence or viral blips were observed between people who did and did not inject drugs. No difference in the rate of viral suppression was found between people who did and did not report use of substance drugs or alcohol.

## Discussion

During the study period, recreational drug use increased among MSM who were followed from acute infection through up to 10 years of ART in our cohort in Bangkok, Thailand. Recruits from 2017 to 2019 had higher rates of substance use overall as well as different use patterns. More recent recruits reported less frequent use of erectile dysfunction drugs and poppers, but more use of methamphetamine, both smoked and injected.

Methamphetamine has become cheaper and more available in Southeast Asia, with the price decreasing by approximately two-thirds over the last decade, even as incomes in the region have risen [1]. This time period coincides with more frequent reports of chemsex, or hi-fun, among MSM in middle- and high-income countries [8].

In Thailand, “ice (i.e., crystal methamphetamine) parties” are an urban phenomenon among young MSM, and have become popular with the spread of dating applications and online social networks [18]. High-risk practices, including unprotected sexual intercourse with multiple partners and episodes of sexual violence, are common during ice parties. MSM participating in ice parties often underestimate the health risks associated with hi-fun, reporting that their primary concern is avoiding arrest and punitive state laws [19].

There was a dramatic increase in injection drug use observed among participants enrolled after 2017. Injection drug use among Thai MSM almost exclusively occurs in the context of hi-fun and group sex parties, and is usually limited to methamphetamine injection [12]. In fact, none of the participants in the cohort reported heroin injection. Moreover, sharing of needles was denied by all of the injection drug users, indicating a high level of knowledge about safe injecting practices and the ability to access safe injecting equipment. The sexual health harm caused by drug injection in the context of chemsex appears to be mediated through a disinhibitory effect on sexual behavior, which may lead to an increased number of sex partners, condomless sex, and rougher or more traumatic sexual practices [20].

Recreational drug use, and methamphetamine use in particular, has been identified as a risk factor for HIV infection among MSM in Asia [5]. However, fewer data are available about the harmful effects of substance use on Asian MSM living with HIV. Chemsex is also known to increase transmission of STIs [21]. Consistent with the literature, in our cohort, recreational drug use was associated with rising incidence of HCV infection and STIs, mediated by riskier sexual behavior such as participation in group sex activities.

With the exception of alcohol, the most commonly reported frequency of use for each substance was less than once a month at any time point during the observation period. Very few cohort participants reported substance use on a weekly or daily basis. The low frequency of substance use, along with use in the context of social gatherings such as group sex parties, may indicate that few participants suffered from addiction or practiced substance use frequently and severely enough to affect their mental health.

Previous studies have reported that substance use is associated with higher viral load and a progressive decline of CD4 T-cells, particularly among participants with heavy alcohol use or problematic drug use [22], and that viral suppression was achieved only after a period of abstinence or reduction of illicit drug use [23]. Recreational drug use in our cohort was associated with more frequent missed doses of ART and more frequent viral blips. However, reduced adherence to ART among substance users in the cohort did not translate into adverse immunologic outcomes or virologic rebound, including among injection drug users.

A previous study among Thai people living with HIV (PLWH) also reported no association between substance use, viral suppression, and ART adherence [24]. People who use recreational drugs in Western HIV cohorts often have other conditions that contribute to poor medication adherence and adverse clinical outcomes, including addiction, mental health issues, and low socioeconomic status [25]. By contrast, most participants enrolled in the present study had a higher level of education and higher household income compared with the Thai general population [17]. They also reported infrequent substance use, most commonly monthly or less, indicating low levels of addiction. These observations suggest that drug use itself does not lead to unsuppressed viral load or poor clinical outcomes for PLWH in the absence of other psychosocial risk factors, especially in a cohort of participants on more recent ART regimens, in which treatment failure is known to be rare [26].

Injection use of methamphetamine among MSM in Bangkok is an emerging trend that carries greater risk for blood-borne disease transmission and adverse health outcomes than other routes of substance use. Although we found no significant associations between injection drug use and immunologic or virologic outcomes of ART, the power to detect associations may have been limited by the recency of the phenomenon and the relatively low number of participants who reported drug injection.

There are additional limitations to the analysis. Participants in the RV254/SEARCH010 study were predominantly young Thai MSM who acquired HIV in geographic areas around Bangkok and Pattaya, Thailand. These findings might not be generalizable to other populations, such as older people, women, or PLWH who start ART in more advanced stages of immunosuppression or in other geographic areas. Moreover, the updated and more comprehensive questionnaire on substance use was introduced in 2017, limiting full comparisons with earlier time periods. MSM living with HIV might also have underreported substance use data because of reporting bias or fear of judgment by the clinical staff. However, reporting biases should have been minimized by implementing the substance use questionnaires in a self-completed online format.

A comprehensive harm-reduction approach is needed to address the problem of drug use among Thai MSM, both HIV-infected and -uninfected [27]. Harm reduction consists of interventions aimed at minimizing health and safety issues associated with drug use and conducted without any form of judgment, coercion, or discrimination, including physical/sexual health, mental health, and sociolegal services [28]. Punitive policies such as the Thai government's “War on Drugs” launched in 2003 and focused on injection heroin users have never been shown to be effective in reducing substance use [29]. Community-based programs in both high- and in low- and middle-income countries promote evidence-based harm reduction, including interventions such as syringe exchange, linkage to substance use disorder treatment facilities, peer-delivered naloxone, and drug checking [30]. Our findings highlight the need to expand harm-reduction programs beyond opiate drug users and individuals who meet the clinical criteria for addiction, since much of the drug use observed in our cohort was episodic in the social context of hi-fun parties among MSM with high socioeconomic status.

It should be noted that the questionnaire used in this study did not inquire about the specific practice of chemsex and was implemented only recently, in 2017, representing a limitation of the study.

Substance use screening at diagnosis and at every routine visit should be integrated into routine clinical practice for MSM with HIV in long-term care in Thailand. For those who screen positive, counseling for harm reduction could potentially mitigate the adverse clinical outcomes.

## Declarations of competing interest

The authors have no competing interests to declare.

## Appendix Supplementary materials

Image, application 1
