# Supplementary material for: Chemsex and rising substance use linked to sexually transmitted infections among men who have sex with men living with HIV in Bangkok, Thailand
Source: IJID Reg. 2024 Sep 28;13:100465. doi: 10.1016/j.ijregi.2024.100465 (PMC11525466; doi:10.1016/j.ijregi.2024.100465)
Supplement: Supplementary file 1 [file mmc1.docx]

**Supplemental Table 1. Sexually transmitted infections and sexual behavior associated with alcohol and recreational drug use**

|  | **N** | **Substance use, N (%)** | **OR (95%CI)** | **p-value** |
| --- | --- | --- | --- | --- |
| **AUDIT C ≥ 4** |  |  |  |  |
| Hepatitis C incidence |  |  |  |  |
| No | 545 | 205 (37.6) | Ref. |  |
| Yes | 59 | 25 (42.4) | 1.22 (0.71 – 2.10) | 0.475 |
| Syphilis incidence |  |  |  |  |
| No | 491 | 182 (37.1) | Ref. |  |
| Yes | 113 | 48 (42.5) | 1.25 (0.83 – 1.90) | 0.286 |
| Gonorrhea prevalence |  |  |  |  |
| No | 468 | 173 (37.0) | Ref. |  |
| Yes | 131 | 55 (42.0) | 1.23 (0.83 – 1.83) | 0.296 |
| Chlamydia prevalence |  |  |  |  |
| No | 437 | 158 (36.2) | Ref. |  |
| Yes | 162 | 70 (43.2) | 1.34 (0.93 – 1.94) | 0.115 |
| Group sex |  |  |  |  |
| No | 438 | 152 (34.7) | Ref. |  |
| Yes | 166 | 78 (47.0) | 1.67 (1.16 – 2.40) | 0.006 |
| **Recreational drug use** |  |  |  |  |
| Hepatitis C incidence |  |  |  |  |
| No | 545 | 240 (44.0) | Ref. |  |
| Yes | 59 | 43 (72.9) | 3.42 (1.88 – 6.21) | <0.001 |
| Syphilis incidence |  |  |  |  |
| No | 491 | 208 (42.4) | Ref. |  |
| Yes | 113 | 75 (66.4) | 2.69 (1.75 – 4.13) | <0.001 |
| Gonorrhea prevalence |  |  |  |  |
| No | 468 | 206 (44.0) | Ref. |  |
| Yes | 131 | 75 (57.3) | 1.70 (1.15 – 2.52) | 0.008 |
| Chlamydia prevalence |  |  |  |  |
| No | 437 | 191 (43.7) | Ref. |  |
| Yes | 162 | 90 (55.6) | 1.61 (1.12 – 2.31) | 0.010 |
| Group sex |  |  |  |  |
| No | 438 | 150 (34.3) | Ref. |  |
| Yes | 166 | 133 (80.1) | 7.74 (5.04 – 11.89) | <0.001 |
| **Methamphetamine use** | **N** | **Substance use, N (%)** | **OR (95%CI)** | **p-value** |
| Hepatitis C incidence |  |  |  |  |
| No | 545 | 102 (18.7) | Ref. |  |
| Yes | 59 | 27 (45.8) | 3.66 (2.10 – 6.39) | <0.001 |
| Syphilis incidence |  |  |  |  |
| No | 491 | 88 (17.9) | Ref. |  |
| Yes | 113 | 41 (36.3) | 2.61 (1.67 – 4.08) | <0.001 |
| Gonorrhea prevalence |  |  |  |  |
| No | 468 | 84 (18.0) | Ref. |  |
| Yes | 131 | 43 (32.8) | 2.23 (1.45 – 3.45) | <0.001 |
| Chlamydia prevalence |  |  |  |  |
| No | 437 | 84 (19.2) | Ref. |  |
| Yes | 162 | 43 (26.5) | 1.52 (1.00 – 2.32) | 0.052 |
| Group sex |  |  |  |  |
| No | 438 | 55 (12.6) | Ref. |  |
| Yes | 166 | 74 (44.6) | 5.60 (3.69 – 8.50) | <0.001 |
| **Methamphetamine injection** |  |  |  |  |
| Hepatitis C incidence |  |  |  |  |
| No | 545 | 33 (6.1) | Ref. |  |
| Yes | 59 | 13 (22.0) | 4.38 (2.16 – 8.91) | <0.001 |
| Syphilis incidence |  |  |  |  |
| No | 491 | 32 (6.5) | Ref. |  |
| Yes | 113 | 14 (12.4) | 2.03 (1.04 – 3.94) | 0.037 |
| Gonorrhea prevalence |  |  |  |  |
| No | 468 | 28 (6.0) | Ref. |  |
| Yes | 131 | 16 (12.2) | 2.19 (1.14 – 4.18) | 0.018 |
| Chlamydia prevalence |  |  |  |  |
| No | 437 | 27 (6.2) | Ref. |  |
| Yes | 162 | 17 (10.5) | 1.78 (0.94 – 3.36) | 0.075 |
| Group sex |  |  |  |  |
| No | 438 | 5 (1.1) | Ref. |  |
| Yes | 166 | 41 (24.7) | 28.40 (10.99 – 73.41) | <0.001 |
